# Clinical application of diffusion tensor tractography in classification of pediatric optic chiasmatic gliomas: a retrospective cohort study

**DOI:** 10.3389/fped.2026.1789704

**Published:** 2026-06-03

**Authors:** Ping Yang, Yuting Liang, Jiashu Chen, Zhuo Zhi, Wei Yang, Jia Wang, Chen Liang, Xiaojiao Peng, Yingjie Cai, Yuanqi Ji, Wenping Ma, Ming Ge

**Affiliations:** 1 Department of Neurosurgery, Beijing Children’s Hospital Capital Medical University, Beijing, China; 2 Department of Pediatrics, Beijing Haidian Maternal and Child Health Hospital, Beijing, China

**Keywords:** brain tumor, diencephalic syndrome, diffusion tensor tractography, optic chiasmatic gliomas, prognosis factors

## Abstract

**Background:**

The classification based on diffusion tensor tractography (DTT) has already been applied to the assessment of the optic chiasmatic glioma (OCGs), but the value of clinical application remains controversial. This study aimed to evaluate the predictive value of DTT classification for clinical phenotypes and to investigate the prognostic factors in children with OCGs.

**Methods:**

A total of 53 patients with OCGs diagnosed and admitted between July 2015 and November 2022 were included. Clinical parameters were collected from medical records. The prognosis analysis comprised clinical, endocrinologic, ophthalmologic and pathologic during different time spans. On DTT images, the tumors were divided into the inflating endophytic type, the inflating exogenous type, and the infiltrative endophytic type based on DTT features. Regression analysis detected the evaluation of classifications which was assessed by determining age, gender, weight, diencephalic syndrome diagnosis record, vision-related data, endocrine-related indicators, surgery related indicators, survival data, chemotherapy, and radiotherapy.

**Results:**

We observed that the proportion of males in the inflating exogenous type patients (10%) was significantly lower than other two types. The inflating endophytic type patients (median 9.5 kg, 4.2∼25.4 kg) had lower body weight at surgery than other two types, as well as higher prevalence of diencephalic syndrome (DS). Postoperative chemotherapy demonstrated a statistically significant protective effect on OS and EFS (*p* = 0.032 and 0.017, respectively).

**Conclusion:**

The DTT-based classification effectively identifies the inflating endophytic subtype, which is associated with a high incidence of diencephalic syndrome, and serves as a valuable tool for preoperative surgical guidance. Combined adjuvant chemotherapy represents a decisive therapeutic strategy that significantly prolongs long-term prognosis in pediatric OCG patients.

## Introduction

Optic pathway gliomas (OPGs) refer to astrocytomas originating from the optic nerve pathway (optic nerve, optic chiasm and optic tract), accounting for approximately 3%–5% of pediatric craniocerebral tumors (1), which are predominantly classified as low-grade pilocytic astrocytoma. Approximately 75% of all cases of optic pathway glioma will develop clinical symptoms in the first decade of life (1), and its early clinical manifestations are related to the primary site of the tumor. Typical symptoms may include visual impairment, painless exophthalmos, visual field defects, nystagmus, and symptoms related to intracranial hypertension. Most OPGs are sporadic, but a small number of children are associated with neurofibromatosis type 1 (NF1) (2). Some researchers believe that NF1-related optic pathway gliomas are more likely to invade the optic nerve, which also have better prognosis (3). OPGs occur in different locations, which are named based on the location of the tumor. OPGs with primary lesions located in the optic chiasm are called optic chiasmatic gliomas (OCGs).

Historically, the Dodge classification (DC) proposed in 1958 (4) and its 2008 revision, the Modified Dodge classification (MDC) (5), have been used to guide surgical resection and prognosis assessment of OPGs. However, the complex three-dimensional anatomical relationship between the tumor and the optic nerve cannot be adequately and intuitively revealed by DC and MDC. To address this, diffusion tensor tractography (DTT), based on magnetic resonance diffusion tensor imaging (MRDTI), has emerged as a valuable tool. DTT can intuitively visualize target white matter fiber bundles and adjacent areas to optimize clinical evaluation and surgical strategies. In 2015, Ge et al. proposed a novel classification of OCGs based on the DTT feature of tumors, which divided the OCGs into two distinct types: inflating type and the infiltrative endophytic type (3), which was thought to be efficient in guiding the surgical treatment. However, while DTT classification optimizes local surgical management, the comprehensive treatment of OCGs often requires a multidisciplinary approach, including adjuvant therapies, to achieve long-term disease control. The clinical benefits of different therapeutic modalities, particularly chemotherapy, remain highly variable and warrant further investigation. Therefore, in this retrospective study, we compared the baseline data to evaluate the effectiveness of this new classification system and to explore the relevant factors affecting the long-term prognosis of children with OCGs.

## Methods

### Patients and methods

We retrospectively reviewed the patients with optic pathway gliomas primarily located in the optic chiasm diagnosed and admitted to the Department of Neurosurgery at our hospital, between July 2015 and November in 2022. Inclusion criteria were age <18 years at diagnosis. Patients were initially identified through a retrospective search of our institutional electronic medical record database using diagnostic keywords related to optic pathway and chiasmatic tumors. The diagnosis of OPG was established based on the presence of an optic pathway space-occupying lesion on preoperative cranial contrast-enhanced MRI, combined with postoperative histopathological confirmation. Exclusion criteria included unclear imaging findings and incomplete medical records. All patients were serially evaluated from the clinical, endocrinologic, ophthalmologic and pathology. All clinical factors being investigated were collected through a system of access to medical records by surgeons. Contrast-enhanced MRI was routinely performed at 1 week, 3 months, 6 months, 9 months, 12 months，24 months and 36 months after surgery to determine whether the tumor recurred. Follow-up data were obtained from outpatient follow-up and telephone follow-up every 6 months. Preoperative and postoperative visual function test results were collected by ophthalmologists from children with OCGs referred to the ophthalmology clinic.

Patients underwent routine tumor resection or biopsy and received modified German International Society for Pediatric Oncology low-grade glioma 2004 regimen chemotherapy after surgery. This study protocol was approved by the Medical Ethics Committee of the hospital. This study was in accordance with the principles of the Declaration of Helsinki. All patients/participants provided their written informed consent to participate in this study. Research study protocols were approved by patients and their parents. Parents provided written informed consent to participate. This study was approved by the Medical Ethics Committee of our hospital (REC approval number: 2022-E-065-Y). Since this article is a retrospective clinical study, it has not registered as a clinical trial.

### DTT examination parameters

All imaging data were obtained on a 3.0T MRI scanner (Siemens Trio Tim, Germany) with an 8-channel head coil and sequence signal acquisition via interleaved echo planar imaging from 30 diffusion gradient directions. The specific parameters includes: FOV 256 mm,TR/TE 11000/94 ms, Slice thickness/gap 2.0/0 mm, EPI factor 128, voxel size 2.0 × 2.0 × 2.0 mm, Band width 1502 Hz/Px, and scan time duration 6 min 16 s.

### Image processing methods

Image processing was conducted using manually outlined regions of interests (ROIs). A scanner workstation equipped with Neuro 3D image processing software (3.0T Siemens Trio Tim, Germany) was used to draw the bilateral optic nerves, optic chiasm, optic tract, and optic radiation. Specifically, images were extracted from the T1-MPRAGE scanning sequence, and multiple seed regions were selected in both tumor tissue and adjacent normal tissue in the bilateral optic nerves and optic chiasm on the axial images. Fractional anisotropy threshold 0.05, curvature threshold 308. The preoperative DTT images were independently evaluated and classified by two experienced neuroradiologists who were blinded to the clinical baseline. Any discrepancies in the DTT classification between the two evaluating neuroradiologists were resolved through a joint consensus discussion. If a consensus could not be reached initially, a senior pediatric neurosurgeon was included in the discussion to make the final determination. We calculated the Cohen's Kappa coefficient to assess the inter-observer agreement with blind to the clinical data. The Kappa value was 0.82, indicating substantial agreement.

**Figure 1:**
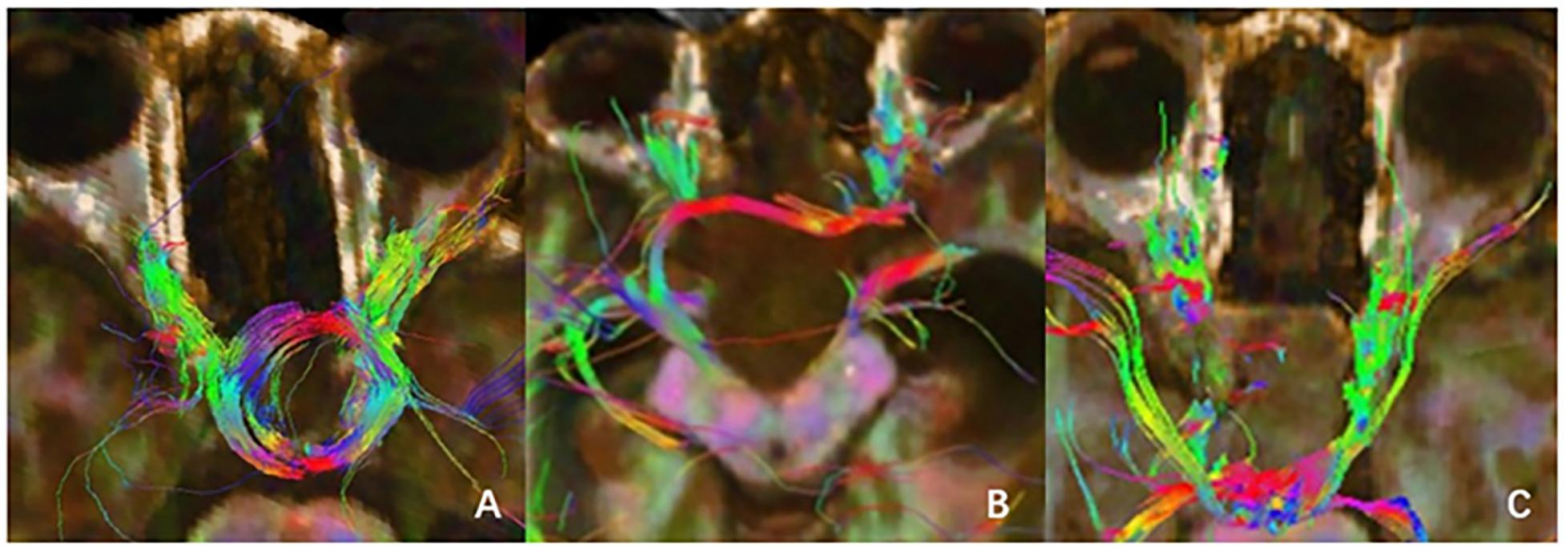
Examples of the three types. **(A)** The inflating endophytic type: the residual optic chiasm fiber bundles diverged around the tumors; **(B)** The inflating exogenous type: the visualized optic chiasm fiber bundles exhibited trajection in the tumors and interrupted continuity in certain fiber bundles; **(C)** The infiltrative endophytic type: the optic chiasm was fused to the tumor, which could not be distinguished from normal structures using in DTT images.

According to the relationship between optic chiasm fiber bundles and the position of the tumors visualized by DTT, the patients were divided into three types: the inflating endophytic type, the inflating exogenous type, and the infiltrative endophytic type. The inflating endophytic type was defined as the residual optic chiasm fiber bundles diverged around the tumors; the inflating exogenous type was defined as the visualized optic chiasm fiber bundles exhibited trajection in the tumors and interrupted continuity in certain fiber bundles; the infiltrative endophytic type defined as the optic chiasm was fused to the tumor, which could not be distinguished from normal structures using in DTT images. Examples of the three types are presented in Figure 1.

### Evaluation of outcomes

The data of the base line were obtained from the medical records. The evaluation indicators included the age at surgery, gender, weight, diencephalic syndrome diagnosis record, vision-related data, endocrine-related indicators, surgery-related indicators, survival data, chemotherapy, and radiotherapy. According to the “Growth and Development Standards for Children under 7 years of Age” and “Grade Evaluation of height Development of children and Adolescents aged 7–18 years” issued by the Chinese Health Commission (http://www.nhc.gov.cn), the children were divided into underweight (weight < −2SD), standard weight (−2SD weight ≤ +2SD), and overweight (weight > +2SD). Diencephalic syndrome (DS) is characterized by failure to thrive, emaciation, hyperkinesia, despite normal or slightly decreased caloric intake, and normal linear growth.

Vision-related data included preoperative functional vision examination and postoperative functional vision changes. Patients underwent functional vision examination before and one week after surgery. Functional vision impairment was defined as the inability to count fingers, no hand motion perception, or no light perception according to the visual examination. Preoperative and postoperative functional vision was assessed by the youngest patient using observation (to observe the child's response to light source, face, moving object) and occlusion testing (to cover one eye and observe the child' s ability to follow the object).

Endocrine-related data included postoperative hormone levels, diabetes insipidus and long-term medication. One week after surgery, the enrolled patients received, endocrine examinations including serum ACTH, TT3, TT4, TSH, FT3, and FT4 levels. Serum electrolytes were measured twice a day for at least 3 consecutive days after surgery once abnormal serum electrolytes were defined as postoperative electrolyte disorder.

Surgical-related indicators included pathological diagnosis, surgical approach, intraoperative blood loss, and extent of surgical resection. The extent of surgical resection was determined according to postoperative enhanced MRI images. All the enrolled patients received tumor resections by three senior neurosurgeons. Approaches were chosen based on their clinical and radiological manifestations, including the preoperative DTT results. The extent of resection was defined as: near-total resection (more than 90% tumor resection), subtotal resection (70%–90% tumor resection), partial resection (less than 70% tumor resection), and biopsy (only performed needle biopsy under neuron navigation). Tumors with the extent of surgical resection was determined by the relationship between optic chiasm fiber and the position of the tumors visualized by DTT, as well as the actual intraoperative situation.

Event-free survival (EFS) was selected as the primary endpoint for statistical analysis. And overall survival (OS) was defined to be the secondary endpoint. OS was defined as the time between the first surgical diagnosis of OCGs and the death of the child. EFS was defined as the time from surgery to any event: radiographic progression (an increase in lesion size vs. the first postoperative MRI), new lesion development, distant metastasis, symptomatic deterioration, or death. Patients without observed tumor progression were considered event-free until the end of follow-up.

### Statistical analysis

Categorical variables are expressed as frequencies and proportions, while continuous variables are presented as medians [interquartile ranges (IQR)]. For comparison of differences between groups, Fisher's exact test was used for categorical variables and the Kruskal–Wallis test was used for continuous variables. Kaplan–Meier method was used for survival analysis. The prognostic factors included age, gender, DTI-based classification, diagnosis of the diencephalic syndrome, NF-1, long-term exogenous hormone supplementation, the extent of surgical resection, radiotherapy, and chemotherapy. Univariate analysis was performed by log-rank test, and multivariate analysis was performed by Cox proportional hazards regression. All factors with *p* < 0.15 in univariate analysis were included in multivariate analysis. In all analyses, *p* < 0.05 was considered statistically significant. All analyses were performed using SPSS (version 25.0, SPSS Inc, IBM, Armonk, NY, USA).

## Results

According to the inclusion and exclusion criteria, a total of 53 patients were included in the study. Among all patients, 33 (62.3%) were inflating endophytic type, 10 (18.9%) were inflating exogenous type and 10 (18.9%) were infiltrative endophytic type. Of the 53 patients, 23 were male and 30 were female, with a median age of 1.79[1.01–4.24] years. NF-1 mutation was present in 13 patients (24.5%). The baseline data of the patients are presented in Table 1.

**Table 1:** Comparison of baseline clinical characteristics among the three types.

Baseline characteristics	The inflating endophytic type	The inflating exogenous type	The infiltrative endophytic type	*p* value
Gender				0.024
Male	15 (45.5%)	1 (10%)	7 (70%)	
Female	18 (54.5%)	9 (90%)	3 (30%)	
Age of onset (years)	1.74[0.79–3.72]	3.41[0.99–5.41]	3.98[1.22–7.38]	0.126
Weight (kg)	9.50[6.45–15.00]	13.00[9.25–18.80]	19.00[9.80–31.10]	0.033
Weight grouping of age				0.042
Underweight	22 (66.7%)	3 (30%)	3 (30%)	
Standard weight	10 (30.3%)	7 (70%)	7 (70%)	
Overweight	1 (3.0%)	0	0	
Diencephalic syndrome				0.013
(+)	22 (66.7%)	2 (20%)	3 (30%)	
(−)	11 (33.3%)	8 (80%)	7 (70%)	
NF-1				0.656
(+)	9 (27.3%)	1 (10%)	3 (30%)	
(−)	24 (72.7%)	9 (90%)	7 (70%)	
Pathologic diagnosis				0.303
Pilocytic astrocytoma	23 (69.7%)	6 (60%)	7 (70%)	
Pilomyxiod astrocytoma	9 (27.3%)	4 (40%)	1 (10%)	
Diffuse astrocytoma	1 (3.0%)	0	1 (10%)	
Oligodendroglioma	0	0	1 (10%)	
Radiotherapy				1.00
(+)	3 (9.1%)	0	0	
(−)	30 (90.9%)	10 (100%)	10 (100%)	
Chemotherapy				0.556
(+)	23 (30.3%)	7 (70%)	9 (90%)	
(−)	10 (69.7%)	3 (30%)	1 (10%)	
Median follow-up time	25.73[9.68–56.28]	30.90[15.65–54.93]	19.18[6.18–42.23]	

Statistical analysis showed that there were differences in gender among the three types of patients(*p* = 0.024). The proportion of males in the inflating exogenous type patients (10%) was significantly lower than in the inflating endophytic type patients (45.5%) and the infiltrative endophytic type patients (70%). Among 30 female patients, the proportion of females in the inflating endophytic type patients (60%) was higher than in the inflating exogenous type patients (30%) and the infiltrative endophytic type patients (10%).

The inflating endophytic type patients (9.50[6.45–15.00]kg) had lower body weight at surgery than the inflating exogenous type patients (13.00[9.25–18.80]kg) and the infiltrative endophytic type patients (19.00[9.80–31.10]kg) (*p* = 0.033). After adjustment for age, the inflating endophytic type patients still had higher rate of underweight (66.7% vs. 30%, 30%, *p* = 0.042). The prevalence of diencephalic syndrome was also higher in the inflating endophytic type patients (66.7%) than in the inflating exogenous type patients (20%) and the infiltrative endophytic type patients (30%) (*p* = 0.013).

There were four pathological diagnoses in 53 patients, including pilocytic astrocytoma, pilomyxiod astrocytoma, diffuse astrocytoma, and oligodendroglioma. And no difference was between the three types (*p* = 0.303). In regards to postoperative radiotherapy (*p* = 1.00) and chemotherapy (*p* = 0.556), no statistically significant difference was found among the three groups of patients.

### Vision and neuroendocrine result

Preoperative and postoperative functional vision changes are presented in Table 2. The analysis showed no difference in preoperative functional vision level (*p* = 0.126) and postoperative functional vision change (*p* = 0.775) among different types of patients.

**Table 2:** Comparison of vision-related data among the three types.

Vision	The inflating endophytic type	The inflating exogenous type	The infiltrative endophytic type	*p* value
Preoperative vision				0.126
Normal	18 (54.5%)	4 (40%)	2 (20%)	
Impairment	15 (15.5%)	6 (60%)	8 (80%)	
Postoperative vision				0.775
Better	1 (3.0%)	1 (10%)	0	
The same	12 (36.4%)	4 (40%)	4 (40%)	
Worse	20 (60.6%)	5 (50%)	6 (60%)	

The postoperative neuroendocrine profile is presented in Table 3. Hypothyroxinemia was present in 6(18.2%) patients with the inflating endophytic type, but not in those with the inflating exogenous type or the infiltrative endophytic type. However, there was no significant difference in hypokalemia among the three types of patients (*p* = 0.541). There were also no significant differences in cortisol levels, incidence of electrolyte disturbance (*p* = 0.151), and incidence of postoperative diabetes insipidus (*p* = 0.431) among the three types of patients.

**Table 3:** Comparison of endocrine-related data among the three types.

Postoperative Endocrine levels	The inflating endophytic type	The inflating exogenous type	The infiltrative endophytic type	*p* value
Thyroid level				0.541
Normal	27 (81.8%)	10 (100%)	10 (100%)	
Low	6 (18.2%)	0	0	
Cortisol levels				0.570
Normal	28 (84.8%)	9 (90%)	7 (70%)	
Low	5 (15.2%)	1 (10%)	3 (30%)	
Electrolyte disturbance				0.151
Absent	4 (12.1%)	2 (20%)	4 (40%)	
Present	29 (87.9%)	8 (80%)	6 (60%)	
Diabetes insipidus				0.431
Absent	9 (27.3%)	2 (20%)	5 (50%)	
Present	24 (72.7%)	8 (80%)	5 (50%)	

### Surgery

28 (84.8%) of the inflating endophytic type patients and all the 10 (100%) inflating exogenous type patients had resection of ≥70%. 3 (30%) of the infiltrative endophytic type patients underwent needle biopsy. Statistical analysis showed a significant difference in the extent of resection among patients with different types (*p* = 0.014). In terms of the choice of surgical approach, the anterior interhemispheric approach was the most used surgical approach and was chosen in 23 (69.7%), 7 (70%), and 5 (50%) patients. There are differences in the choice of surgical approach among patients with different types (*p* = 0.046), but there was no difference in intraoperative blood loss (*p* = 0.887). The surgery-related data is presented in Table 4.

**Table 4:** Comparison of surgery-related data among the three types.

Surgery related indicators	The inflating endophytic type	The inflating exogenous type	The infiltrative endophytic type	*p* value
Extent of surgical resection				0.014
≥70%	28 (84.8%)	10 (100%)	6 (60%)	
＜70%	5 (15.2%)	0	1	
Needle biopsy	0	0	3 (30%)	
Surgical approach				0.046
Anterior interhemispheric approach	23 (69.7%)	7 (70%)	5 (50%)	
Transcallosal interforniceal approach	9 (27.3%)	2 (20%)	2 (20%)	
Transcortical approach	0	1 (10%)	3 (30%)	
Needle biopsy	0	0	0	
Subfrontal approach	1 (3%)	0	0	
Intraoperative blood loss				0.887
＜200mL	25 (75.8%)	8 (80%)	9 (90%)	
≥200mL	8 (8%)	2 (20%)	1 (10%)	

### Analysis of survival

**Figure 2:**
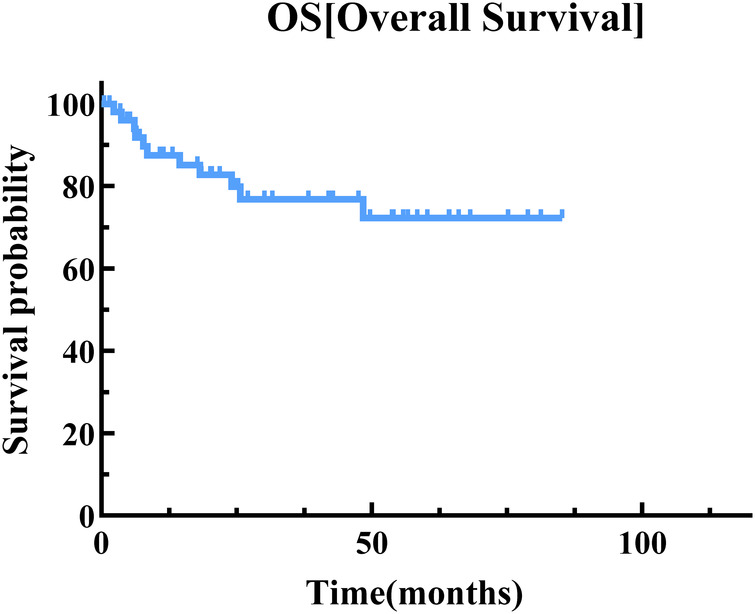
OS curves of 53 OPG patients.

**Figure 3:**
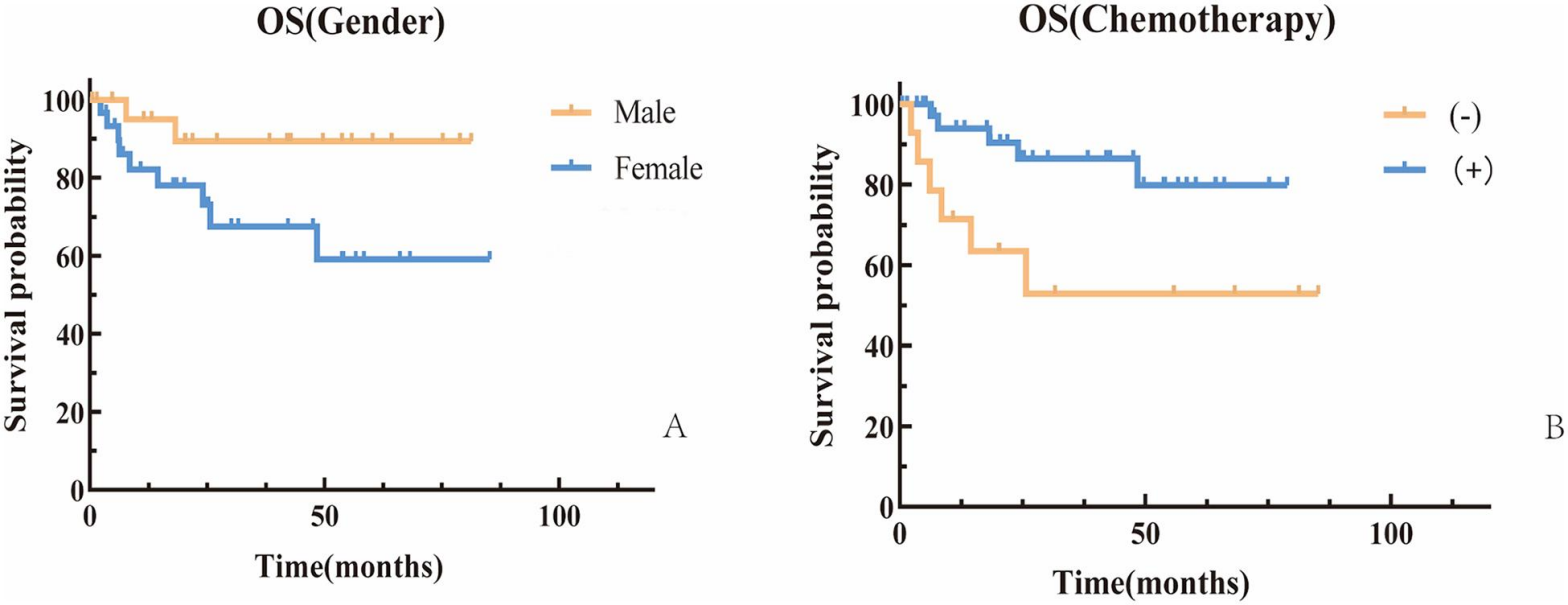
Independent risk factors of OS based on log-rank test. **(A)** OS based on gender. **(B)** OS based on receiving chemotherapy or not.

The median follow-up time of 53 patients was 25.27[9.68–53.88] months and the average OS of 53 patients was 66.7 months. 11 (21.8%) patients died, and 42 (79.2%) patients survived. The 5-year overall survival (OS) rate was 81.1% (Figure 2). The results of the univariate survival analysis are shown in Table 5. The results of univariate analysis showed that the average OS of male (74.10 months) was higher than that of female (58.62 months) (*p* = 0.062). In terms of surgery, the average OS of patients with extent of resection ≥70% (68.51 months) was significantly higher than that of patients with degree of resection <70% (13.71 months), however, all three patients who underwent biopsy were alive at the time of the last follow-up (*p* = 0.108). The average OS was longer in patients who received chemotherapy after surgery (68.26 months) than in those who did not receive chemotherapy (50.42 months) (Figure 3).

**Table 5:** Univariate analysis of OS in 53 OPG patients.

Factors	Numbers	Average OS(M)	*P* value
Age(years)			0.906
＜2	27	61.06	
≥2	26	67.74	
Gender			0.062
Male	23	74.10	
Female	30	58.62	
DTI-based classification			0.479
The inflating endophytic type	33	62.55	
The inflating exogenous type	10	60.10	
The infiltrative endophytic type	10	53.26	
Diencephalic syndrome			0.810
(+)	27	67.55	
(−)	26	62.82	
NF-1			0.698
(+)	11	50.20	
(−)	42	66.12	
Long-term exogenous hormone supplementation			0.847
(+)	16	50.84	
(−)	37	67.33	
Extent of surgical resection			0.108
＞70%	45	68.51	
≤70%	5	13.71	
Needle biopsy	3		
Radiotherapy			0.876
(+)	3	64.83	
(−)	50	62.54	
Chemotherapy			0.022
(+)	39	68.26	
(−)	14	50.42	

There was no statistically significant difference in the average OS of the inflating endophytic type patients (62.55 months), the inflating exogenous type patients (60.10 months) and the infiltrative endophytic type patients (53.26 months) (*p* = 0.479).

Multivariate analyses of OS are presented in Table 6. Our results indicated that postoperative chemotherapy served as a protective factor to improve OS for children with OPG (*p* = 0.032).

**Table 6:** Multivariate analysis of OS in 53 OPG patients.

Factors	*β*	RR	95%CI	*P* value
Female	–	–	–	0.128
Age ＜2 year	–	–	–	0.508
NF-1	–	–	–	0.902
Diencephalic syndrome	–	–	–	0.645
DTT-based classification	–	–	–	0.602
The inflating exogenous type vs. the inflating endophytic type	–	–	–	0.370
The infiltrative endophytic type vs. the inflating endophytic type	–	–	–	0.786
Extent of surgical resection	–	–	–	0.724
＞70% vs. others	–	–	–	
Chemotherapy	1.299	3.665	1.117–12.031	0.032

**Figure 4:**
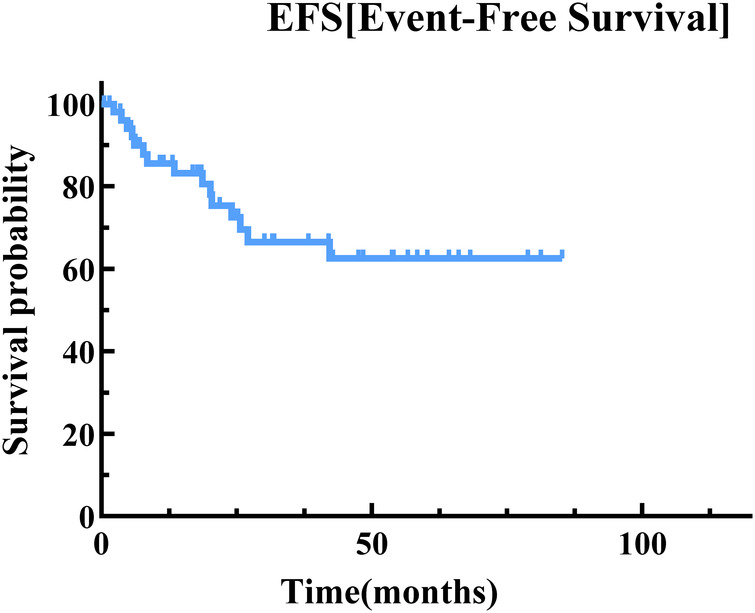
EFS curves of 53 OPG patients.

**Figure 5:**
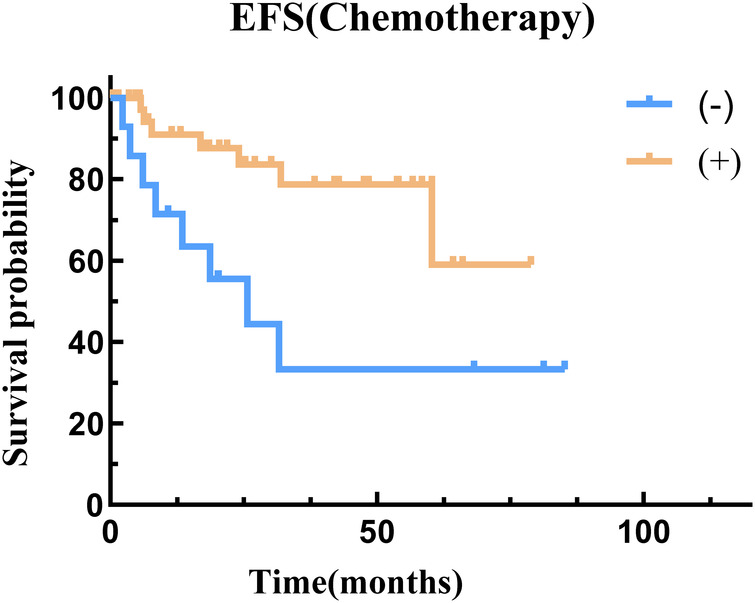
EFS based on receiving chemotherapy or not.

The average EFS of 53 patients was 59.0 months, 15 (28.3%) patients with tumor recurrence (Figure 4). The results of the univariate analysis of EFS in 53 patients are shown in Table 7. The average EFS of patients with extent of resection >70% (59.19 months) was signifi.cantly higher than that of patients with degree of resection ≤70% (18.37 months), but the *p*-value was 0.903. The average EFS was longer in patients who received chemotherapy after surgery (62.16 months) than in those who did not receive chemotherapy (38.79 months) (*p* = 0.011) (Figure 5).

**Table 7:** Univariate analysis of EFS in 53 OPG patients.

Factors	Numbers	Average OS(M)	*P* value
Age(years)			0**.**804
<2	27	54**.**25	
≥2	26	59**.**92	
Gender			0**.**290
Male	23	62**.**51	
Female	30	55**.**39	
DTI-based classification			0**.**462
The inflating endophytic type	33	54**.**36	
The inflating exogenous type	10	53**.**90	
The infiltrative endophytic type	10	53**.**45	
Diencephalic syndrome			0**.**567
(+)	27	56**.**13	
(−)	26	63**.**72	
NF-1			0**.**467
(+)	11	48**.**00	
(−)	42	57**.**37	
Long-term exogenous hormone supplementation			0**.**296
(+)	16	43**.**01	
(−)	37	62**.**71	
Extent of surgical resection			0**.**903
＞70%	45	59**.**19	
≤70%	5	18**.**37	
Needle biopsy	3		
Radiotherapy			0**.**714
(+)	3	64**.**83	
(−)	50	54**.**72	
Chemotherapy			0**.**011
(+)	39	62**.**156	
(−)	14	38**.**793	

Multivariate analyses of EFS are presented in Table 8. Postoperative chemotherapy served as a protective factor to improve EFS for children with OPG (*p* = 0.017).

**Table 8:** Multivariate analysis of EFS in 53 OPG patients.

Factors	β	RR	95%CI	*P* value
Age＜2 year	–	–	–	0.300
NF-1	–	–	–	0.619
DTT-based classification	–	–	–	0.619
The inflating exogenous type vs. the inflating endophytic type	–	–	–	0.501
The infiltrative endophytic type vs. the inflating endophytic type	–	–	–	0.572
Diencephalic syndrome	–	–	–	0.674
Chemotherapy	1.252	3.496	1.253–9.755	0.017

## Discussion

OPGs can be divided into the Dodge (4) and McCullough (6) subtypes, and most OCGs are types II-III within the Dodge subtype. In this study, all 53 OCG patients are III within the Dodge subtype. Miller et al. divided OCGs into pre- and post-chiasmatic types according to the positional relationship between the lesion and the optic chiasm in 1974 (7). There are few studies utilizing DTT in OCGs. In 1990, Wisoff et al. divided OCGs into ingrowth and outgrowth types based on whether the tumor broke through the perineurium into the subarachnoid space (6). However, the existing classification system has limited application in the surgical treatment of OCGs. Ge (3) revised the typing in Wisoff et al. (6) as infiltrative endophytic type (Type I) and the inflated type (Type II) by using DTT images in 2015, which is instructive in the surgical treatment of OCGs. On this basis, we revise as the inflating endophytic type, the inflating exogenous type and the infiltrative endophytic type to the perioperative assessment of OCGs provides a more adequate and intuitive basis for classifying these tumors. In this study, the main tumor body of most patients locate in the optic chiasm. Most OCGs gradually push the remaining optic chiasm structure downward as the tumor grows. Thus, the residual optic chiasma fiber bundles either diverge around the lesion or cross the lesion. DTT classification provides a deeper understanding of OCGs, as well as contributes to improving surgical strategy. It is important to maintain the integrity of the normal optic chiasm structure to retain vision and to reduce the complications of OCGs in the surgical treatment (8, 9). The aim of this study was to further investigate the clinical value of the novel DTT-based classifications of OPGs and identify factors influencing prognosis.

In our cohort, males were significantly less common in the inflating exogenous subtype, while the inflating endophytic subtype was the most prevalent among females. Accordingly, univariate analysis showed a longer average OS for males (74.10 vs. 58.62 months). Although our univariable analysis only revealed a marginally significant trend for gender regarding OS (*p* = 0.062), this trend is consistent with previous literature reporting that female NF1-OPG patients are more prone to tumor progression and visual impairment (10–12). Mechanistically, this sex-specific disparity is linked to the estrogen–IL-1β–cAMP axis: estrogen prompts tumor-associated glial cells to act as a “toxic factory” releasing IL-1β to accelerate retinal ganglion cell death and vision loss (13, 14). Notably, while NF-1 mutations were present in 24.5% of our cohort, their distribution across the three DTT subtypes was homogeneous. This critically indicates that the female predilection for the inflating endophytic pattern is driven by this estrogen-mediated “toxic microenvironment” rather than baseline genetic imbalances.

In addition, the study showed that the inflating endophytic type had lower body weight at surgery than the other two types, along with a higher prevalence of DS and worse postoperative vision, which may be caused by different tumor growth patterns of tumors. DS is a rare pediatric condition associated with OPGs (15), which characterized by failure to thrive, emaciation, hyperkinesia, dispite normal or slightly decreased caloric intake, and normal linear growth. Currently, the mechanism underlying DS are still poorly understood and may include increased energy expenditure (16), paradoxically elevated growth hormone levels in response to glucose loads, partial GH resistance and excessive *β*-lipotropin (a lipolytic peptide) secretion, resulting in increased lipolysis of subcutaneous adipose tissue (17). It is possible that the expending endogenous tumor is surround by nerve fibers and grows expansively inside the nerve fibers, which pushed the bilateral optic nerves to each side. And the gradually expanding tumor compress the pituitary gland resulting in anterior pituitary disorders (APDs) (15), which affect the secretion of growth hormone (GH).

The location of OPGs precludes complete surgical resection due to the risk of incurring unacceptable neurological and functional consequences which occurs in a particular location. Presently, the strategy of treating OPGs, especially OCGs, has gradually, yet controversially, evolved (18). Most researchers use chemotherapy and/or radiotherapy as the first-lined treatment (19). Surgery is often restricted to unilateral optic nerve tumors causing severe visual loss, or predominantly exophytic and cystic lesions (2). Goodden et al. claimed that surgery has a clear role for diagnosis, tumor control, and relief of mass effect in children with OCGs (20). The primary challenge in safely executing these surgical objectives lies in preoperative planning. Traditional imaging modalities, such as contrast-enhanced CT and MRI, offer diagnostic value but remain insufficient for delineating the complex microanatomy of visual fibers (21). In contrast, DTT provides a distinct advantage by elucidating the anatomical relationship between the tumor and surrounding white matter fiber tracts (22). In our cohort, intraoperative microscopic findings consistently matched preoperative DTT delineations in the 38 patients who underwent ≥70% resection, validating the reliability of this imaging modality.

Crucially, the core clinical value of this validated DTT classification lies in its capacity for preoperative risk aversion and its direct impact on personalized surgical decision-making. In our practice, the surgical paradigm actively shifted based on DTT findings to prioritize functional preservation. For inflating subtypes, where DTT delineated merely displaced but structurally continuous optic tracts, surgeons were guided through a safe surgical corridor (often via an anterior interhemispheric approach) to pursue maximum safe resection and achieve rapid physical decompression. This approach aligns with the protective strategies advocated by Goodden et al. Conversely, for the infiltrative endophytic subtype, where DTT visualized the deep engulfment or disruption of visual fibers, aggressive debulking was proactively abandoned. Recognizing that extensive resection in this scenario would inevitably cause irreversible postoperative blindness, the surgical team utilized DTT as a navigational safeguard. The procedure was actively restricted to a stereotactic biopsy or limited partial resection. The objective shifted strictly toward obtaining tissue for molecular diagnosis and relieving acute intracranial hypertension, followed by an immediate transition to systemic chemotherapy.

By strictly adhering to this DTT-guided risk aversion strategy, we protected the optic nerves as much as possible while achieving highly competitive clinical outcomes. In our 53-patient cohort, the 5-year OS was 81.1%, and 41.5% of patients maintained stable or improved visual acuity postoperatively. These outcomes are comparable to previous literature. For instance, Hidalgo et al. (23) reported a 10-year OS of 81% and a combined 37% rate of stable or improved vision in patients receiving surgical treatment alone. Although more than 50% of our patients experienced postoperative visual decline, these findings underscore the inherent fragility of the visual pathway. They further highlight why utilizing DTT to avoid overzealous resection is absolutely critical. Given the inherent limitations of a single-center retrospective study, further prospective randomized controlled trials are warranted to definitively establish the superiority and efficacy of this DTT-based classification over existing frameworks.

Furthermore, we found that postoperative chemotherapy demonstrated a statistically significant protective effect on both OS and EFS (*p* = 0.032 and *p* = 0.017, respectively), highlighting its substantial clinical benefit. This finding is consistent with previous cohort studies. For instance, the carboplatin and vincristine (CV) regimen has proven effective in 66.67% of newly diagnosed and 50% of relapsed pediatric OPGs (24). Similarly, utilizing an 18-month CV cycle, the European Association of Pediatric Oncology reported a 5-year progression-free survival (PFS) of 46% and an OS of 89%, with the addition of etoposide providing no further survival prolongation (25). However, it should be noted that while chemotherapy improves survival, it has a limited effect on visual recovery, with only 14.4%–19% of children experiencing improvements in visual acuity or visual fields (26, 27). Therefore, we believe that chemotherapy is highly suitable as a first-line treatment for clinically pediatric OPGs, or as a routine adjuvant therapy following conservative tumor resection.

### Limitation

There is no doubt that this study has several limitations. First, regarding the diagnostic methodology and clinical assessment, the DTT-based classification inherently relies on the subjective selection of ROIs. So, we conducted an inter-observer reliability analysis to maximize objectivity. Additionally, formal visual acuity testing was largely unfeasible due to the young age of our cohort. We relied on observation and cover tests, which, despite lacking absolute precision, remain the most reliable markers for gross visual function in the youngest patients. Second, the associations observed between DTT classifications and certain clinical variables (e.g., gender, weight) were exploratory in nature. Because multiple statistical tests were performed without alpha adjustment, the possibility of chance findings (Type I error) cannot be entirely ruled out. Future prospective studies with predefined hypotheses are required to validate these clinical phenotypes. Finally, our survival analyses did not account for the impact of emerging treatment paradigms. Modalities such as proton therapy or targeted molecular therapies for tumors harboring BRAF mutations were not included in the statistical models, which could significantly alter the morbidity and mortality landscape for this subset of patients. Comprehensive long-term follow-up—incorporating longitudinal DTT imaging, visual outcomes, and updated treatment protocols—is currently lacking. Future large-scale, multicenter prospective studies with predefined hypotheses are warranted to validate these clinical phenotypes and prognostic frameworks.

## Conclusion

It is feasible and will be beneficial to utilize the classifications based on DTT images to diagnosis and treat children with OCGs. The application of the classifications to the peri- operative care of children with OCGs is valuable for increasing our understanding of the disease, developing reasonable surgical strategies. The classifications can empower neurosurgeons to develop personalized, risk-stratified surgical strategies. Combined adjuvant chemotherapy represents a decisive therapeutic strategy that significantly prolongs long-term survival in pediatric OCG patients.

## Data Availability

The datasets presented in this article are not readily available due to patient confidentiality and institutional restrictions. Requests to access the datasets should be directed to Ping Yang, 2814593207@qq.com.
